# Supplementary material for: Reduction of De Novo Lipogenesis Mediates Beneficial Effects of Isoenergetic Diets on Fatty Liver: Mechanistic Insights from the MEDEA Randomized Clinical Trial
Source: Nutrients. 2022 May 23;14(10):2178. doi: 10.3390/nu14102178 (PMC9143579; doi:10.3390/nu14102178)
Supplement: Supplementary file 1 [file nutrients-14-02178-s001.zip › nutrients-1685106-Figure S1.pdf]

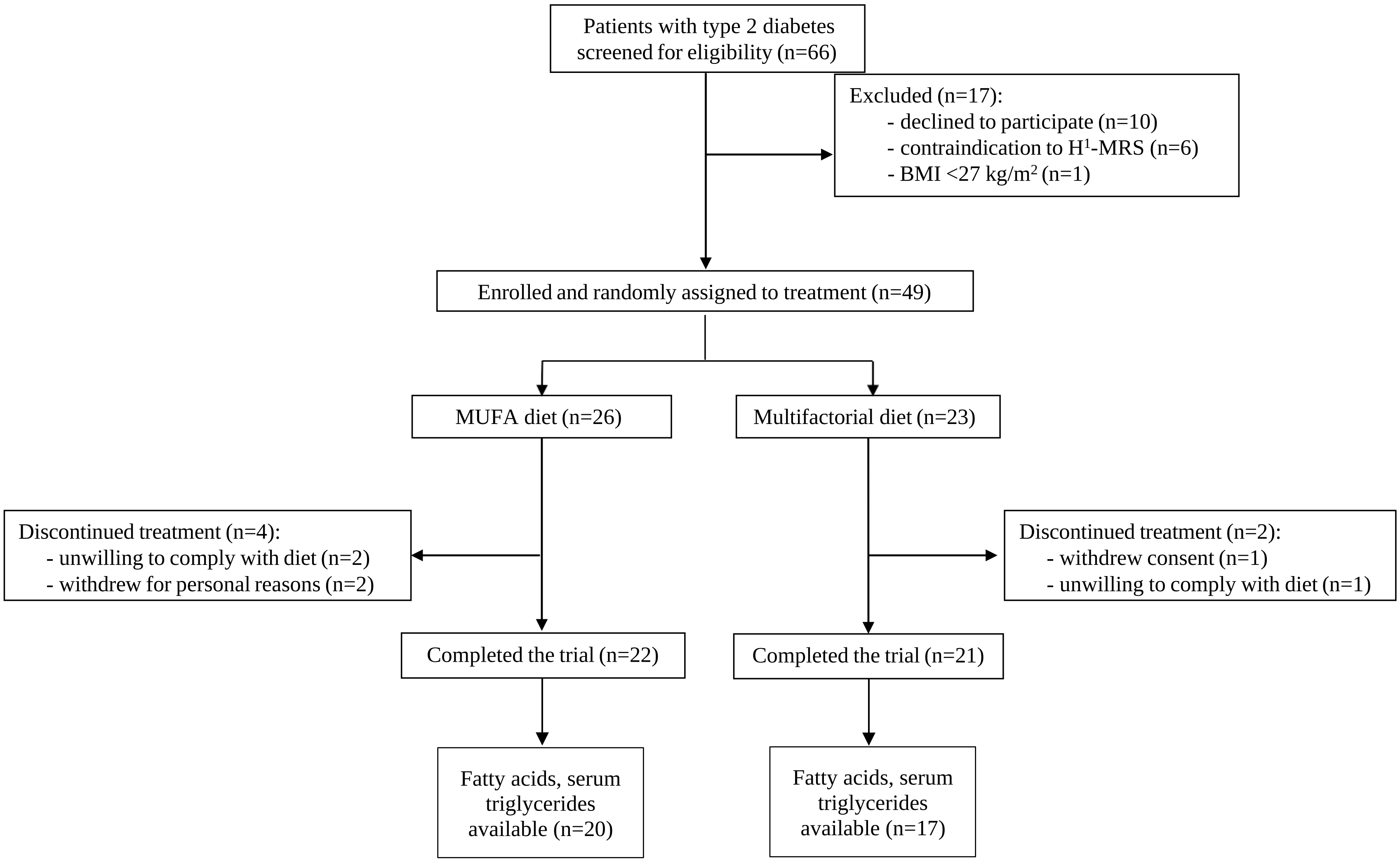
Patients with type 2 diabetes screened for eligibility (n=66)
Excluded (n=17):
- declined to participate (n=10)
- contraindication to H1-MRS (n=6)
- BMI <27 kg/m2 (n=1)
Enrolled and randomly assigned to treatment (n=49)
MUFA diet (n=26)
Multifactorial diet (n=23)
Discontinued treatment (n=4):
- unwilling to comply with diet (n=2)
- withdrew for personal reasons (n=2)
Discontinued treatment (n=2):
- withdrew consent (n=1)
- unwilling to comply with diet (n=1)
Completed the trial (n=22)
Completed the trial (n=21)
Fatty acids, serum triglycerides available (n=20)
Fatty acids, serum triglycerides available (n=17)
